# Prevalence and Phenotypic and Molecular Characterization of Carbapenemase-Producing Gram-Negative Bacteria in Gabon

**DOI:** 10.4269/ajtmh.22-0168

**Published:** 2022-12-19

**Authors:** Annicet-Clotaire Dikoumba, Richard Onanga, Hélène Jean-Pierre, Marie-Noelle Didelot, Yann Dumont, Abdoul-Salam Ouedraogo, Edgard-Brice Ngoungou, Sylvain Godreuil

**Affiliations:** ^1^ Hôpital d’Instruction des Armées Omar Bongo Ondimba, Libreville, Gabon;; ^2^ Centre Interdisciplinaire de Recherches Médicales de Franceville, Franceville, Gabon;; ^3^ Laboratoire de Bactériologie, Centre Hospitalier Universitaire de Montpellier, Montpellier, France;; ^4^ Maladies Infectieuses et Vecteurs: Ecologie, Génétique, evolution et Contrôle, Institut de Recherche pour le Développement, Centre National de la Recherche Scientifique, Université de Montpellier, Montpellier, France;; ^5^ Department of Medical Bacteriology and Virology, National Reference Laboratory for Antimicrobial Resistance, University Hospital Centre Sanou Sourou, Bobo Dioulasso, Burkina;; ^6^ Jeune Equipe Associée à Institut de Recherche pour le Développement, Résistance aux Antimicrobiens au Burkina Faso, Montpellier, France;; ^7^ Département d’Epidémiologie, Biostatistiques et Informatique Médicale/Unité de Recherche en Epidémiologie des Maladies Chroniques et Santé Environnement, Faculté de Médecine, Université des Sciences de la Santé, Libreville, Gabon

## Abstract

Data collection and monitoring of carbapenemase-producing (CP) Gram-negative bacteria (GNB) are often limited. This study determined CP-GNB prevalence in Gabon and the genetic origins of the resistance genes. From January 2016 to March 2018, 869 clinically significant GNB isolates from inpatients and outpatients, and 19 fecal samples (inpatients) were analyzed in the main hospitals of Gabon. Fecal samples were screened using ChromID^®^ CARBA SMART selective chromogenic medium biplates. Species were identified by matrix-assisted laser desorption ionization–time of flight mass spectrometry. Antibiotic susceptibility was tested using the disk diffusion method on Müller–Hinton agar, and resistance genes were assessed by multiplex polymerase chain reaction and sequencing. Overall, 1.61% of clinical isolates (14 of 869) and 5.26% of fecal samples (1 of 19) were CP-GNB. The CP-GNB rate was higher among inpatients (2.98%) than outpatients (0.33%), in intensive care units (28.57%, 4 of 14), and in urine samples (35.71%, 5 of 14). The most common CP-GNB were *Klebsiella pneumoniae* (53.33%) and *Acinetobacter baumannii* (26.67%). *bla*_OXA-48_ was the predominant carbapenemase-encoding gene (40%), followed by *bla*_NDM-5_ (33.33%). The *A. baumannii* multilocus sequence types ST2 and ST78, *Enterobacter cloacae* ST78, *Escherichia coli* ST2, and *K. pneumonia* ST48 and ST147 were found. These data indicate that CP bacteria are present in clinical and carriage samples. Preventive measures are needed to avoid the spread of resistance genes.

## INTRODUCTION

The emergence and spread of carbapenemase-producing (CP) Gram-negative bacteria (GNB) represent a serious public health problem because effective therapeutic alternatives (e.g., colistin and cefiderocol) are not widely available in low-income countries.^1^^–^^3^ Therefore, carbapenems remain often the last available antibiotic class to treat infections caused by multidrug-resistant nonfermenter (*Acinetobacter baumannii* and *Pseudomonas aeruginosa*) and fermenter (*Enterobacterales*) GNB.^4^^,^^5^

Acquired class A (*KPC, IMI, GES*), class B (*IMP, VIM, NDM*), and class D (*OXA-48, OXA-181*) carbapenemases are the most prevalent determinants of resistance to carbapenems.^6^^,^^7^ The supporting genes are associated frequently with mobile genetic structures (e.g., plasmids, integrons, transposons) that promote their spread and transmission. CP-GNB spreading is considered an urgent threat because high mortality rates have been reported among infected patients.^8^^,^^9^ In 2017, the WHO included carbapenem-resistant *Enterobacterales*, *A. baumannii*, and *P. aeruginosa* in the global priority list of antibiotic-resistant pathogens.^10^

CP-GNB have been detected worldwide,^11^ but data collection and surveillance of multidrug-resistant GNB are often limited, particularly in middle- and low-income countries, such as many African countries. In Central Africa, only a few studies have reported the presence of isolates that produce carbapenemases, such as *NDM-4* in Cameroon, *OXA-181* and *NDM-1* in Angola, and *OXA-181* and *NDM-5* in Chad.^4^^,^^12^ In Gabon, only one study described the presence of *NDM-7*-producing isolates.^13^

We investigated the prevalence and genetic characteristics of CP-GNB isolates from clinical and fecal carriage samples from in- and outpatients in Gabon.

## MATERIALS AND METHODS

### Study area, patients, and specimen collection.

**Figure 1.**
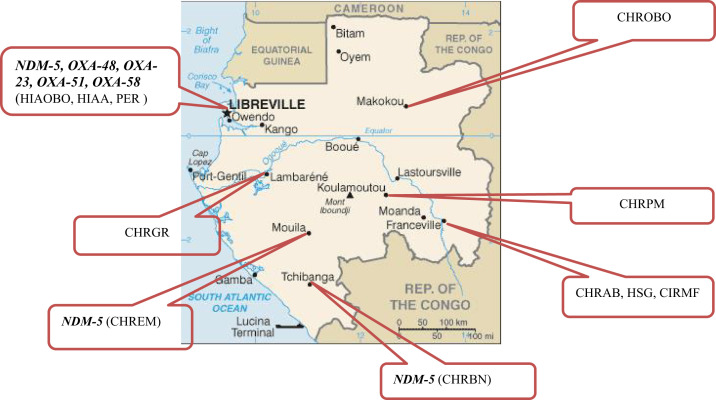
Location of the hospitals in Gabon and carbapenemases (*NDM-5*, *OXA-48*, *OXA-23*, *OXA-51*, and *OXA-58*) produced by the carbapenemase-producing Gram-negative bacteria isolates detected at the indicated hospitals. CHRAB = Center Hospitalier Régional Amissa Bongo; CHRBN = Center Hospitalier Régional Benjamin Ngoubou; CHREM = Center Hospitalier Régional de Mouila; CHRGR = Center Hospitalier Régional Georges Rawiri; CHROBO = Center Hospitalier Régional Omar Bongo Ondimba; CHRPM = Center Hospitalier Régional Paul Moukambi; CIRMF = Center Interdisciplinaire de Recherches Médicales de Franceville; HIAA = Hôpital d’Instruction des Armées d’Akanda; HIAOBO = Hôpital d’Instruction des Armées; HSG = Hôpital Sino-Gabonais; PER = Polyclinique El Rapha. Source: Ambassade du Gabon au Sénégal, 2013. *Informations Générales sur le Gabon*. Available at: http://www.amba-senegal.ga/163-services-aux-usagers/167-informations-generales-sur-le-gabon/#.XyXwzjWhS00. Accessed October 22, 2022.

Samples were obtained from eight main hospitals and one medical analysis laboratory located in seven of the nine provinces of Gabon (population, 1,811,079 inhabitants among whom more than 93% reside in urban areas) from January 2016 to March 2018 (Figure 1). The eight hospitals are located in Libreville, the capital city that concentrates almost half of this population (49.5%)^14^ and includes the Omar Bongo Ondimba Army Teaching Hospital (HIAOBO), Akanda Army Teaching Hospital, and El Rapha Polyclinic; Lambaréné, which includes the Georges Rawiri Regional Hospital Center; Mouila, which includes the Mouila Regional Hospital Center; Tchibanga, which includes the Benjamin Ngoubou Regional Hospital Center; Koulamoutou, which includes the Paul Moukambi Regional Hospital Center; and Moakokou, which includes the Omar Bongo Ondimba Regional Hospital Center. The Medical Analysis Laboratory of the Interdisciplinary Medical Research Center of Franceville is in Franceville and analyzes samples from two hospitals in Franceville: the Amissa Bongo Regional Hospital Center and the Sino-Gabonese Cooperation Hospital Center. Each hospital has an average of 93 beds (thus, in total, ∼930 beds).

During this period, 869 nonduplicate clinical GNB samples were obtained: 569 (65.5%) from patients hospitalized for more than 48 hours and 300 (34.5%) from outpatients. During the same period, fresh stool samples were collected from 19 patients hospitalized for more than 48 hours without digestive pathology at the Omar Bongo Ondimba Army Teaching Hospital and the Mouila Regional Hospital Center to investigate CP-GNB fecal carriage. Briefly, 0.5 g of each fresh stool sample was suspended in 5 mL sterile saline solution (0.9%), and 100-μL aliquots were plated on ChromID^®^ CARBA SMART selective chromogenic medium biplates (bioMérieux, Marcy-l’Etoile, France), a medium used for the screening of CP *Enterobacterales*.

### Species identification and antimicrobial susceptibility testing.

Bacterial isolates were identified by matrix-assisted laser desorption ionization–time of flight mass spectrometry (Bruker Daltonics, Bremen, Germany). Antimicrobial susceptibility was tested with the disk diffusion method on Müller–Hinton agar. The following antibiotics were tested: amoxicillin, amoxicillin–clavulanic acid, aztreonam, cefepime, cefotaxime (CTX), cefpirome, cefpodoxime, cefoxitin, ceftazidime, cephalothin, moxalactam, piperacillin, piperacillin–tazobactam, ticarcillin, ticarcillin–clavulanic acid, imipenem, nalidixic acid, ciproflox acin, levofloxacin, ofloxacin, amikacin, gentamicin, netilmicin, tobramycin (TOB), fosfomycin, chloramphenicol, tetracycline, and trimethoprim–sulfamethoxazole. The minimum inhibitory concentrations for imipenem, meropenem, doripenem (for *P. aeruginosa*, *A. baumannii*, and *Enterobacterales*) and ertapenem (for *Enterobacterales*) were determined with the Etest method (bioMérieux, Marcy-l’Etoile, France) to detect potential CP-GNB isolates. Results were interpreted according to the European Committee on Antimicrobial Susceptibility Testing guidelines and clinical breakpoints.^15^ Extended spectrum beta-lactamase (ESBL) production was detected with the combined double-disk synergy method.^16^

### Molecular identification of antibiotic resistance genes.

After antimicrobial susceptibility testing of the 869 GNB, and measurement of the minimal inhibitory concentrations for imipenem, meropenem, ertapenem, and doripenem, isolates that displayed resistance or intermediate susceptibility to these carbapenems were analyzed by polymerase chain reaction (PCR) to identify antibiotic resistance determinants.

DNA was extracted from one single colony for each isolate in a final volume of 100 µL of distilled water by incubation at 95°C for 10 minutes, followed by a centrifugation step. The presence of the *bla*_NDM_,* bla*_Oxa48-like_, *bla*_GIM_, *bla*_PER_, *bla*_IMP_, *bla*_VIM_, *bla*_SPM_, *bla*_KPC_, *bla*_DIM_, *bla*_SIM_, *bla*_BIC_, *bla*_AIM_, *bla*_VEB_, *bla*_CTX-M_ (*CTX-M* groups 1, 2, 8, 9, and 25), *bla*_TEM_, *bla*_SHV_, and *bla*_OXA-1-like_, the 16S recombinant RNA methylase genes conferring resistance to aminoglycosides (*armA*,* rmtA*,* rmtB*,* rmtC*, and *rmtD*), and the plasmid-mediated quinolone resistance (PMQR) genes (*qnrA*,* qnrB*,* qnrS*,* qnrC*,* qnrD*,* qepA*,* aac(6′)-Ib-cr*, and *oqxAB*) was assessed by multiplex PCR using a previously published method.^17^ Our strategy did not allow discriminating between the *aac (6′)-Ib* gene, conferring resistance to aminoglycosides, and the *aac (6′)-Ib-cr* gene, conferring resistance to aminoglycosides plus quinolones. DNA samples from ESBL-, carbapenemase-, 16S recombinant RNA methylase- and PMQR-positive clinical isolates from the collection of the Montpellier University Hospital bacterial laboratory (Montpellier, France) (previously characterized phenotypically and molecularly), were used as positive controls. PCR products were visualized after electrophoresis on 1.5% agarose gels containing ethidium bromide (Eurobio Scientific, LES ULIS, France) at 100 V for 90 minutes. A 100-bp DNA ladder (Promega, Charbonnières-les-bains, France) was used as marker size. PCR products were purified using the ExoSAP-IT PCR Product Clean-up Reagent (GE Healthcare, Piscataway, NJ USA), and was sequenced bidirectionally on a 3100 ABI Prism Genetic Analyzer (Applied Biosystems). Nucleotide sequence alignment and analyses were performed online using the BLAST program available at the National Center for Biotechnology Information (www.ncbi.nlm.nih.gov).

### Conjugation experiments.

Mating experiments were performed using the azide-resistant *Escherichia coli* strain J53 as recipient cells. In case of transfer failure, plasmid DNA was extracted with the GeneJET Plasmid Miniprep Kit^18^ and transferred into *E. coli* DH10B cells (Invitrogen, Cergy-Pontoise, France).^19^ Plasmids ware characterized by plasmid relaxase gene typing and PCR-based replicon typing.^20^

### Molecular epidemiology typing.

To determine the phylogenetic group of the *E. coli* CP-GNB isolates, the PCR-based method described by Clermont et al.^21^ was used. Multilocus sequence typing (MLST) analysis was performed as described at the Institut Pasteur MLST using whole-genome MLST databases (http://bigsdb.pasteur.fr/) for *E. coli*, *K. pneumoniae*, *Enterobacter cloacae* and *A. baumannii*.

### Statistical analysis.

CP-GNB frequencies were compared between men and women, and then between inpatients and outpatients using the *χ*^2^ test. Statistical analyses were performed using the R version 4.0.2 (R Foundation for Statistical Computing, Vienna, Austria). Differences were considered significant at the 0.05 confidence level.

## RESULTS

### CP-GNB occurrence.

Species identification indicated that the CP-GNB isolates (clinical and carriage) were mainly *K. pneumoniae* (53.33%, 8 of 15), followed by *A. baumannii* (26.67%, 4 of 15), *E. coli* (13.33%, 2 of 15), and *E. cloacae* (6.67%, 1 of 15) (Table 1).

**Table 1:** Characteristics and resistance genes in the CP-GNB clinical and carriage isolates

Origin	Strain code	Bacterial species	Carbapenemase genes	ESBL	Other β-lactamase genes	PMQR genes	16S RNA methylase genes	Phylogroup (*Escherichia coli*)	Plasmid	Sequence type
Clinical	Ab10h1	*Acinetobacter baumannii*	*OXA_23/OXA-51*	–	–	–	*armA*	–	–	2
Clinical	Ab1h11	*A. baumannii*	*OXA-51/OXA-58*	–	–	–	–	–	–	78
Clinical	Ab1h7	*A. baumannii*	*OXA_23/OXA-51*	–	–	–	–	–	–	78
Clinical	Pf1h1	*A. baumannii*	*OXA-23/OXA-51/ OXA-58*		–	–	–	–	–	2
Clinical	En1h14	*Enterobacter cloacae*	*NDM-5*	–	*TEM-1/OXA-1*	*qnrB/aac(6′)-Ib-cr*	–	–	IncX1	78
Clinical	Ec12c57	*Escherichia coli*	*NDM-5*	–	*TEM-1*	–	–	A	IncFIA	2
Carriage	Ko13h6	*E. coli*	*NDM-5*	*CTX-M15*	*TEM-1/OXA-1*	*aac(6′)-Ib-cr*	–	A	IncFIA	2
Clinical	Kp1h37	*Klebsiella pneumoniae*	*NDM-5*	*CTX-M15*	*TEM-1*	*qnrB/aac(6′)-Ib-cr*	–	–	IncX1	48
Clinical	Kp1h38	*K. pneumoniae*	*NDM-5*	*CTX-M15*	*TEM-1*	*qnrB/aac(6′)-Ib-cr*	–	–	IncX1	48
Clinical	Kp1h14	*K. pneumoniae*	*OXA-48*	*CTX-M15*	*OXA-1*	*qnrB/aac(6′)-Ib-cr*	–	–	IncFIIK	147
Clinical	Kp1h17	*K. pneumoniae*	*OXA-48*	*CTX-M15*	*OXA-1*	*qnrB/aac(6′)-Ib-cr*	–	–	IncFIIK	147
Clinical	Kp1h21	*K. pneumoniae*	*OXA-48*	*CTX-M15*	*TEM-1*	*qnrB/ aac(6′)-Ib-cr*	–	–	IncFIIK	147
Clinical	Kp1h27	*K. pneumoniae*	*OXA-48*	*CTX-M15*	–	*qnrB*	–	–	IncFIIK	147
Clinical	Kp1h9	*K. pneumoniae*	*OXA-48*	–	–	*qnrB*	–	–	IncFIIK	147
Clinical	So1h3	*K. pneumoniae*	*OXA-48*	–	–	*aac(6′)-Ib-cr*	–	–	IncX1	147

*aac* = aminoglycoside acetyltransferase resistance gene; CP-GNB = carbapenemase-producing Gram-negative bacteria; *CTX-M* = cefotaximase beta-lactamase gene; ESBL = extended-spectrum beta-lactamase; *NDM* = New Delhi metallo-beta-lactamase gene; *OXA* = oxacillinase gene; PMQR = plasmid mediated quinolone resistance; *qnr* = quinolone resistance gene; *TEM* = temoneria beta-lactamase gene.

Among the 14 clinical CP-GNB isolates, 13 were isolated in hospitals in Libreville, and one in Tchibanga. The only CP-GNB isolate from fecal samples (carriage screening) was isolated in Mouila (Figure 1, Table 2).

**Table 2:** CP-GNB distribution according to type of sample, locality, hospital department, and gender

Sample	City, *n*			Hospital unit, *n*						Ambulatory, *n*	Gender, *n*		Total, *n*
	Libreville	Mouila	Tchibanga	Intensive care	Emergency	Medicine	Cardiology	Neonatology	Neurosurgery		Men	Women	
Urinary catheter	2	0	0	1	0	0	1	0	0	0	2	0	2
Urine	5	0	1	0	1	1	1	0	2	1	3	3	6
Protected distal specimen	1	0	0	1	0	0	0	0	0	0	0	1	1
Wound	0	0	0	0	0	0	0	0	0	0	0	0	0
Blood	5	0	0	2	0	0	0	3	0	0	1	4	5
Anal swab	0	1	0	0	0	1	0	0	0	0	0	1	1
Total	13	1	1	4	1	2	2	3	2	1	6	9	15

CP-GNB = carbapenemase-producing Gram-negative bacteria.

Among the 14 clinical CP-GNB isolates, 14.29% (2 of 14) were isolated from urinary catheter, 35.71% (5 of 14) from urine, 7.14% (1 of 14) from a protected distal specimen, none from a wound, and 35.71% (5 of 14) from blood culture samples (Table 2). CP-GNB isolates were detected more frequently in samples from intensive care units (28.57%, 4 of 14), followed by neonatology (21.43%, 3 of 14); cardiology, neurosurgery, and internal medicine (14.29%, 2 of 14); and emergency services (7.14%, 1 of 14) (Table 2).

Overall, CP-GNB were detected in 1.61% of clinical samples (14 of 869), with six samples from male patients and eight samples from female patients (*P* = 0.76), and 13 samples from inpatients and one sample from an outpatient (*P* = 0.03) (Table 3); and in 5.26% of fecal carriage samples (1 of 19).

**Table 3:** CP-GNB and NCP-GNB distribution according to patient gender and type

Bacteria	Men, *n*	Women, *n*	*P* value	Inpatients, *n*	Outpatients, *n*	*P* value
CP-GNB	6	9	0.76	14	1	0.03
NCP-GNB	464	390	–	555	299	–
Total	470	399	–	569	300	–

CP-GNB = carbapenemase-producing Gram-negative bacteria; NCP-GNB = noncarbapenemase-producing Gram-negative bacteria.

Statistical differences were assessed with the *χ*^2^ test.

### Antibiotic susceptibility profiles.

In addition to resistance to third- and fourth-generation cephalosporins, antimicrobial susceptibility testing of the 15 CP-GNB isolates (*n* = 14 clinical samples and *n* = 1 carriage sample) showed high rates of resistance to aminoglycosides, fluoroquinolones, and trimethoprim–sulfamethoxazole: 80% (12 of 15) to gentamicin, 86.67% (13 of 15) to tobramycin, 26.67% (4 of 15) to amikacin, 93.33% (14 of 15) to ciprofloxacin, 80% (12 of 15) to levofloxacin, and 80% (12 of 15) to trimethoprim–sulfamethoxazole.

### Molecular characterization of CP-GNB resistance.

Table 1 summarizes the different carbapenemases and associated resistance genes, the phylogroup *E. coli*, and the plasmids carrying the resistance genes detected in the 15 CP-GNB isolates.

The four CP *A. baumannii* isolates carried two or three *OXA*-encoding genes: *bla*_OXA-23_/*bla*_OXA-51_, *bla*_OXA-51_/*bla*_OXA-58_, and *bla*_OXA-23_/*bla*_OXA-51_/*bla*_OXA-58_. Six CP *K. pneumoniae* clinical isolates (40%) carried the *bla*_OXA-48_ gene. Four clinical CP-GNB isolates (26.67%; *n* = 2 *K. pneumoniae*, *n* = 1 *E. cloacae*, and *n* = 1 *E. coli*) carried the *bla*_NDM-5_ gene as well as the CP-GNB fecal isolate (*E. coli*).

Nine CP-GNB isolates (60%) carried one to three beta-lactamase–encoding genes: *bla*_CTX-M15_ (*n* = 7), *bla*_TEM-1_ (*n* = 6), and *bla*_OXA-1_ (*n* = 4). Genes implicated in resistance to quinolones and aminoglycosides, such as *qnrB* and *aac(6′)-Ib-cr* (quinolones; *n* = 10 CP-GNB isolates) and *armA* (aminoglycosides; *n* = 1), were also detected. The *armA*-harboring *A. baumannii* clinical isolate also carried the *bla*_OXA-23_ and *bla*_OXA-51_ genes.

The two *E. coli* isolates (*n* = 1 urinary sample and *n* = 1 fecal sample) belonged to phylogroup A, and the resistance genes were carried by the same plasmid (IncFIA). These isolates were from two patients (one inpatient and one outpatient) at hospitals in two different cities (Mouila and Tchibanga), 170 km apart.

The *bla*_NDM-5_ gene detected in two *K. pneumoniae* isolates from blood cultures of two patients hospitalized in the intensive care unit of HIAOBO (Libreville) was on the IncX1 plasmid. Eleven carbapenemase genes (73.33%) were identified on plasmids belonging to three different incompatibility groups (IncX1, IncFIA, and IncFIIK).

The CP *A. baumannii* strains belonged to the sequence type 2 (ST2) clone (*n* = 2) and ST78 (*n* = 2) clone, and harbored the *bla*_OXA-23/OXA-51_ (*n* = 2), *bla*_OXA-51/OXA-58_ (*n* = 1), and *bla*_OXA-23/OXA-51/OXA-58_ (*n* = 1) genes. The only CP *E. cloacae* strain belonged to the ST78 clone and harbored the *bla*_NDM-5_ gene, whereas the two CP *E. coli* strains belonged to the ST2 clone and harbored the *bla*_NDM-5_ gene. Among the eight CP *K. pneumonia* strains, two belonged to the ST48 clone and harbored the *bla*_NDM-5_ gene, and six belonged to the ST147 clone and harbored the *bla*_OXA-48_ gene.

## DISCUSSION

This study found low rates of CP-GNB in Gabon (1.61% for clinical samples and 5.26% for fecal carriage). The low rate of CP-GNB clinical samples is similar to that reported by a study in Chad (2.5%),^4^ but is less than in Cameroon (Central Africa; 11.11%) and Senegal (West Africa; 5.1%).^22^^,^^23^ Conversely, it is more than that in Burkina Faso (West Africa; 0.9%).^24^ This low rate in Gabon could be explained by the high cost of carbapenems, which reduces the risk of excessive use, and therefore promotes the appearance of resistance. The emergence of resistance to carbapenems in Gabon could be the consequence of the implementation in 2008 of a universal health insurance (Caisse Nationale d’Assurance Maladie et de Garantie Sociale) that facilitates the prescription and purchase of these antibiotics. In 2016, Moussounda et al.^13^ reported the same prevalence (5.1%) of fecal carriage at the HIAOBO military training hospital in Libreville (samples harboring *bla*_OXA-48_ and *bla*_NDM-7_), whereas Sanou et al.^24^ found greater prevalence in Burkina Faso (8.2%). Although the number of fecal samples in our study was very low, our results indicate the presence of CP-GNB carriage in Gabon. This is worrying because it potentially increases the risk of nosocomial and community-acquired CP-GNB infections.

Our study found that CP-GNB prevalence was greater among inpatients (2.98%) than outpatients (0.33%, *P* < 0.05), which is in line with a recent study in Punjab (Pakistan)^25^ that reported CP-GNB rates of 93.8% and 6.2% among inpatients and outpatients, respectively. This finding is the consequence of the antibiotic treatment pressure and of the dissemination of carbapenemase genes in hospitals. Indeed, in our study, 73.33% of carbapenemase genes (11 of 15) were located on plasmids, and 91% of them (10 of 11) were detected in CP-GNB isolates from inpatients, particularly from intensive care (28.57%) and neonatology units (21.43%). The greater risk of nosocomial infections in intensive care units may be a result of the critical conditions and suppressed immunity of patients needing intensive care.^25^

CP-GNB were isolated mainly in urine samples (35.71%), as described previously in the south of France.^26^ Urinary tract infections are among the most frequent human bacterial infections, and in recent years, CP-GNB have often been implicated in them, further complicating their management.^27^

The identified CP-GNP isolates were resistant not only to third- and fourth-generation carbapenems, but also to aminoglycosides and fluoroquinolones. This increases the risk of mortality of patients infected by CP-GNB, particularly in intensive care units, because the available treatment options for intensive care unit–acquired infections resulting from CP-GNB are limited.^28^

The four CP *A. baumannii* isolates carried the *bla*_OXA-23_, *bla*_OXA-51_, and *bla*_OXA-58_ genes. Carbapenems are the most commonly used antibiotics for treating infections caused by *A. baumannii*, and an increase in carbapenem-resistant *A. baumannii* strains has been reported worldwide during the past decade.^29^ In Africa, carbapenem resistance in *A. baumannii* has never been detected in Gabon, but only in South Africa, Libya, Egypt, Tunisia, Algeria, and Senegal.^30^^–^^32^ Interestingly, one *A. baumannii* isolate from urine harbored both the *bla*_OXA-23_ and *bla*_OXA-58_ genes. Their coexpression is rare and was reported first in France in a strain from a urine sample of a patient without previous hospitalization who had traveled to Indonesia.^26^ No patient in our study had a history of travel to Indonesia.

Moreover, five CP-GNB isolates carried the *bla*_NDM-5_ gene, which belongs to group B of the Ambler classification. To our knowledge, this is the first report of its presence in Gabon. *NDM-5* is a variant of the *NDM* carbapenemase that was identified in a multidrug-resistant *E. coli* ST648 isolate from the perineum and throat of a patient in the United Kingdom with history of hospitalization in India.^33^ Since then, *NDM-5* has been detected in other countries, such as Egypt, Algeria, Spain, Japan, Australia, the United States, China, and Chad.^4^^,^^34^ The presence of metallo-beta-lactamases (*NDM-5*) in Gabon can be explained by the trade intensification during the past 10 years that facilitates contacts with Asian countries (India and China), and consequently the dissemination of carbapenemase producers.^35^

The *bla*_OXA-48_ gene is most frequently found in *K. pneumoniae* (six of eight CP *K. pneumoniae* isolates in our study), and has often been reported in Turkey, the Middle East, North Africa, and Europe.^36^ Its detection is less frequent in Central Africa, and was almost nonexistent in Gabon. Similar to *NDM-5*, this could be the consequence of trade intensification. On the other hand, the lack of systematic detection of carbapenemases could hide the beginning of local spreading.

In our study, many CP-GNB isolates also carried other resistances genes, such as beta-lactamase-encoding genes, PMQR genes, and 16S RNA methylase-encoding genes. In Gabon, as a result of the absence of alternative molecules (e.g., colistin or cefiderocol), these resistant isolates are potentially dangerous because carbapenems are still used as “last-line” treatment of infections caused by resistant GNB, including those producing ESBLs.^37^

In addition, one of the CP *A. baumannii* isolates carried the *bla*_OXA-23_, *bla*_OXA-51_, and *armA* genes. In 2015, El-Sayed-Ahmed et al.^38^ reported the coexistence of *bla*_OXA-23_ and *armA* in *A. baumannii* isolates in Egypt. This demonstrates that mobile genetic elements (i.e., plasmids) can carry several different genes, thus facilitating the circulation and dissemination of resistance genes among different bacterial species and genera.

The fact that, in two *K. pneumoniae* isolates from different patients in the same intensive care unit, the *bla*_NDM-5_ gene was located on the same IncX1 plasmid supports the hypothesis of a probable nosocomial infection by the nursing staff.^39^ It also confirms that plasmids are key genetic elements in the dissemination of antimicrobial drug resistance in bacteria,^40^ as suggested by the finding in our study that 73.33% of carbapenemase genes were carried by plasmids.

Among CP-GNB isolates, the sequence type distribution in *Enterobacterales* shows a majority of ST47 (*n* = 6) followed by ST48 (*n* = 2) in *K. pneumoniae*, ST2 in *E. coli* (*n* = 2), and one ST78 in *E. cloacae*. Among the four *A. baumannnii* isolates, two belonged to ST2 and two to ST78.

All the *OXA-48*–producing *K. pneumoniae* strains belonged to the ST147 clone, and were isolated in the same hospital (the Omar Bongo Ondimba Regional Hospital Center military teaching hospital). The clonal dissemination and outbreak of *OXA-48*-producing *K. pneumoniae* were reported in a Chinese hospital in 2016, and *K. pneumoniae* ST147 was among the two predominant clones associated with the outbreak.^41^ In Belgium and Canada, the *K. pneumoniae* ST147 clone has been associated with ESBL and *OXA-48* production.^42^ Like *E. coli* ST410, the *K. pneumoniae* ST147 clone is considered a high-risk nosocomial clone because of its global distribution, association with various antimicrobial resistance genes, prolonged persistence in the host, efficient transmission among hosts, enhanced fitness and virulence, and capacity to cause severe or recurrent infections.^43^

A study in Thailand^44^ revealed the presence of ST147 strains in pets. Because this suggests that such strains can be transmitted between humans and companion animals, it is advisable to put in place public health surveillance measures in veterinary hospitals to minimize infection by multidrug-resistant bacteria in pets. Transmission of ST2, ST48, and ST78 clones is responsible of the spread of drug resistance.^45^^–^^47^

A major limitation of our study is the lack of whole-genome sequencing (WGS) data to characterize our isolates. WGS is the best technology for molecular epidemiology studies of antimicrobial resistance and is used to characterize the genetic basis of resistance mechanisms. However, because of its high cost, complexity, and lack of availability in genetic facilities, WGS is not used routinely in low-income countries such as Gabon.

## CONCLUSION

Our study highlights the production of carbapenemases by clinical, community, and carriage GNB isolates in Gabon. Good hygiene practice should be encouraged to avoid/reduce the spread of resistance genes within hospitals and to reduce nosocomial infections caused by multiresistant bacteria. Moreover, public authorities should implement a real health policy of prevention.
